# Research Advances of the Effects of Food and Medicine Homology Substances on Gastric Cancer

**DOI:** 10.1002/cam4.71242

**Published:** 2025-09-29

**Authors:** Zhengyang Hao, Xiangjun Chen, Wenqian Tang, Ruipeng Wu, Bei Xue, Jingzhe Chen, Yidan Zhang, Shaokang Wang

**Affiliations:** ^1^ Clinical Medical Research Center for Plateau Gastroenterological Disease of Xizang Autonomous Region, and School of Medicine Xizang Minzu University Xianyang China; ^2^ Key Laboratory of Environmental Medicine and Engineering of Ministry of Education, and Department of Nutrition and Food Hygiene, School of Public Health Southeast University Nanjing China

**Keywords:** active ingredients, food and medicine homology substances, gastric cancer, mechanisms of action

## Abstract

**Background:**

Gastric cancer (GC), a common malignant tumor, poses a significant threat to human health. Conventional radiotherapy and chemotherapy regimens come with significant drawbacks, including high toxicity, adverse side effects, inadequate targeting ability, and the potential for developing drug resistance, which ultimately diminishes patients' overall well‐being.

**Methods:**

Food and medicine homology substances exhibit favorable pharmacological activity and minimal toxic side effects. They comprise a range of components that are beneficial to the human body, including high‐quality proteins, vitamins, minerals, and other biologically active components such as polysaccharides, flavonoids, saponins, and alkaloids. In recent years, they have attracted considerable attention in the field of GC prevention and treatment. A comprehensive review of extant literature, alongside a rigorous analysis of contemporary nutrition and traditional Chinese medicine, forms the foundation of this study.

**Results:**

Its systematic approach encompasses the examination of the effects and mechanisms of food and medicine homology substances and their active ingredients on the prophylaxis and therapy of GC.

**Conclusions:**

The study's findings expand the cognitive boundaries of GC prevention and improvement and offer novel insights and directions for the development of clinical anticancer therapeutic drugs.

## 1 Introduction

Gastric cancer (GC) is one of the most prevalent malignant neoplasms worldwide [1, 2], and data from 2022 demonstrate that it possesses the fifth‐highest incidence and mortality rate of all malignant neoplasms [3]. Globally, more than 1 million people are diagnosed with GC each year [4]. Early screening for GC is not a widespread practice, largely because the symptoms of early GC are not readily apparent. Consequently, the detection of early GC remains low, and most patients are not diagnosed until the disease has progressed to an advanced stage. Risk factors for GC include *Helicobacter pylori* ( *H. pylori* ) infection, smoking, and a high salt and fat intake. As demonstrated in studies [5, 6, 7], consuming a diet high in whole grains, fruits, vegetables, and nuts, and low in salt may reduce the likelihood of developing GC. The standard treatment modalities for GC mainly currently encompass radiotherapy and chemotherapy [8], which have the potential to induce adverse effects such as bone marrow suppression, organ damage, and gastrointestinal reactions. It is important to note that some patients may experience poor tolerance, which has the potential to impact the efficacy of the treatment and the patient's quality of life. However, both methods have significant toxic side effects and potential for drug resistance. In recent years, people are increasingly conscious of food safety and health, sparking a surge in demand for nutritious options that offer both health benefits and medicinal value [9].

Food and medicine homology substances refers to naturally occurring substances that can be ingested as foodstuffs and possess specific medicinal properties [10]. On a global scale, there is an increasing interest in the consumption of a healthy diet. In the contemporary era, individuals are seeking sustenance that not only ensures safety and provides nutrients, but also fosters physical and mental well‐being, is environmentally sustainable, is economically equitable and affordable, and respects cultural traditions and dietary habits [11]. In the prevention and improvement of GC, it has significant advantages over traditional radiotherapy and chemotherapy, such as high safety, low toxicity and side effects, and suitability for long‐term use [12]. They are frequently employed within traditional medical systems for disease prevention and treatment, demonstrating significant therapeutic value and nutritional benefits [13, 14]. Apparently, these substances frequently comprise a range of components known to be beneficial to the human body [15]. Such components include proteins, vitamins, minerals, and biologically active components, primarily polysaccharides [16], flavonoids [17], saponins [18], and alkaloids [19]. The natural safety and efficacy of the substance in question have been demonstrated in the prevention and treatment of a wide range of diseases, including Alzheimer disease [20, 21], diabetes mellitus [22, 23], hyperlipidemia [24, 25], obesity [26], hypertension [27, 28], and GC [29].

In this paper, a review of the literature was conducted to summarize the food and medicine homology substances and their active ingredients that play a role in GC. The mechanisms of these substances against GC were then analyzed in terms of antioxidant, anti‐inflammatory, and anti‐*H. pylori*, protection of gastric mucous membrane barrier, immunomodulation, apoptosis, inhibition of value added, and migration of GC cells, respectively. The objective is to provide a reference for the research and development of health products with anti‐GC medicinal and food ingredients.

## 2 Food and Medicine Homology Substances and Their Bioactive Components for the Prevention and Treatment of GC

In recent years, a growing body of research has identified the medicinal properties of numerous foods. These foods have been utilized to enhance human immunity and prevent diseases through dietary therapy, dietary supplementation, or medicinal diets. These findings underscore the dual properties of food and medicine homology substances and have garnered international attention in the field of herbal medicine research due to their low toxicity, high efficiency, and long‐term clinical references [30, 31]. Particularly in the field of tumor management and therapy, the safety and efficacy of food and medicine homology substances are well documented, and these substances have become a novel strategy for cancer treatment, making a significant contribution to human health [32].

**FIGURE 1:**
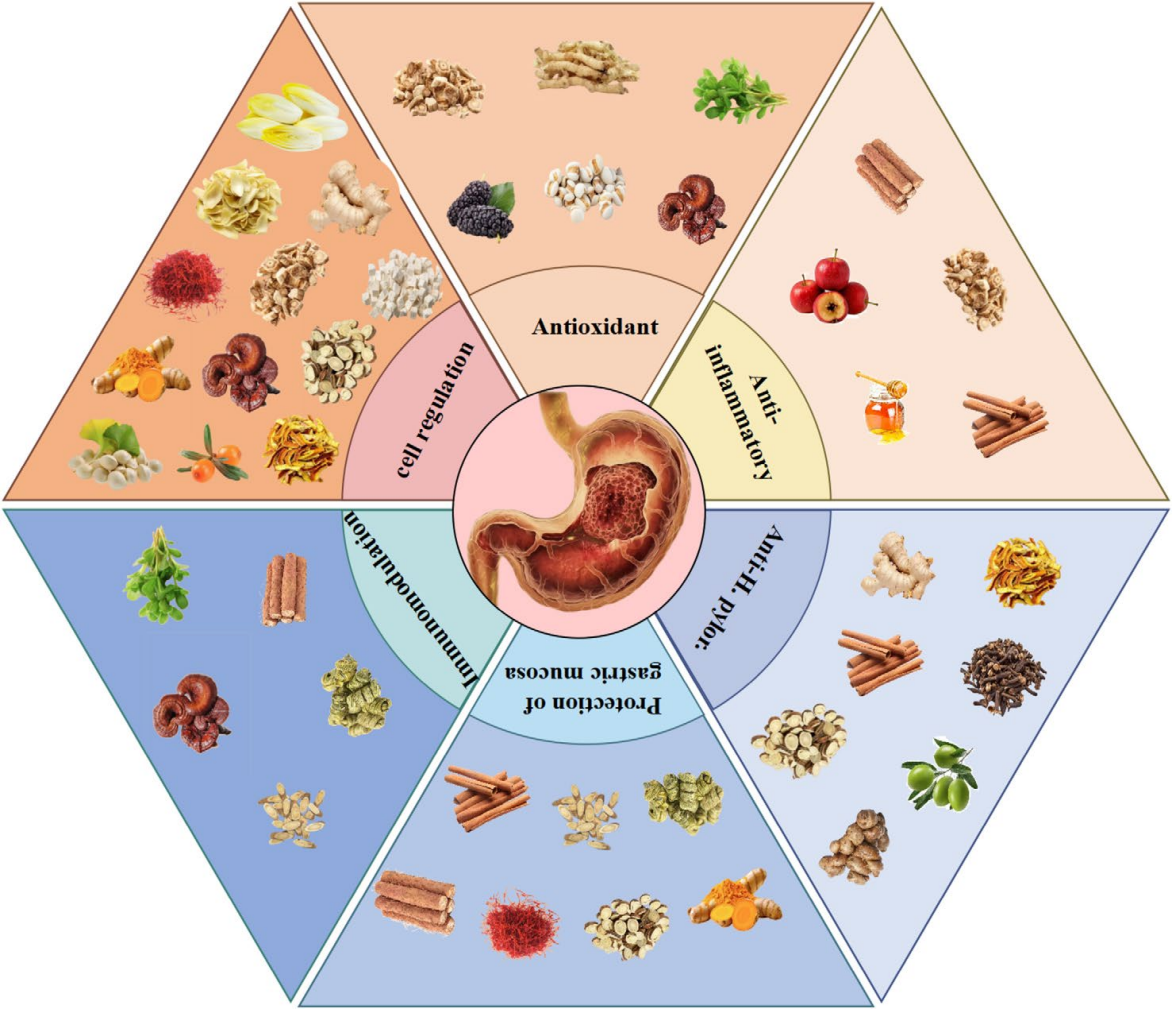
Food and medicine homology substances with preventive and therapeutic effects on GC.

As depicted in Figure 1, food and medicine homology substances used for GC includes ginger, Chinese yam, turmeric, wolfberry, coix seed, licorice, astragalus, and hawthorn. These substances are rich in a variety of active ingredients, such as alkaloids, saponins, and polysaccharides. Their pharmacological mechanisms are mainly reflected in antioxidant, anti‐inflammatory, protecting gastric mucosa, inducing apoptosis of GC cells, inhibiting the proliferation and migration of GC cells, and assisting traditional treatment.

## 3 Mechanism of Action of Food and Medicine Homology Substances and Their Active Ingredients on GC

### 3.1 Antioxidant

Oxidative stress plays a pivotal role in the development and progression of GC [33]. Oxidative stress is defined as a state of imbalance between the production of reactive oxygen species (ROS) and reactive nitrogen species (RNS) and antioxidant capacity [34, 35]. In recent years, a significant body of research has indicated a close relationship between ROS and cancer, with cancer cells being observed to produce higher levels of ROS in comparison to healthy cells [36]. A greater number of antioxidant components are present in food and medicine homology substances, including phenolic compounds and polysaccharides.

Oligofructose (FOS) is a prebiotic that can be extracted from the seeds of *Coix lachryma‐jobi* Linn., and the antioxidant activity of FOS‐containing extracts for 1,1‐diphenyl‐2‐trinitrophenylhydrazine (DPPH) radical scavenging is comparable to that of vitamin C [37]. Resveratrol isolated from mulberry was found to reduce ROS generation, lactate dehydrogenase (LDH) level, and malondialdehyde (MDA) content by increasing catalase (CAT) activity, superoxide dismutase (SOD) activity, and glutathione (GSH) content, which in turn acted as an antioxidant [38]. A review of the biological activities and pharmacological applications of *Polygonatum sibiricum* polysaccharides (PSP) was conducted, which indicated that PSP significantly reduced MDA levels in skeletal muscle and serum, decreased free radical activity, and increased SOD and GSH peroxidase (GSH‐Px) activity [39]. In vitro bioactivity studies have demonstrated that the pectic polysaccharide PGP‐1‐1, extracted from *Platycodon grandiflorus* , can enhance the body's antioxidant function by promoting cellular antioxidant gene expression and restoring the antioxidant defense capacity of hydrogen peroxide (H_2_O_2_)‐treated intestinal cells [40]. A number of studies have demonstrated that Ganoderma lucidum extract and Ganoderma lucidum polysaccharide have antioxidant properties [41, 42]. Ganoderma lucidum polysaccharides can exert antioxidant functions by increasing the activity of antioxidant enzymes, regulating the expression of Bcl‐2, and the ratio of Bax/Bcl‐2 [43]. In addition, they have been shown to enhance the antioxidant activity of N‐methyl‐N9‐nitro‐N‐nitrosoguanidine‐induced GC in rats, achieved by decreasing the levels of IL‐6 and TNF‐α, and increasing the levels of IL‐2, IL‐4, IL‐10, SOD, CAT, and GSH‐Px [44]. Dendrobium officinale polysaccharides have been demonstrated to reduce 8‐OHdG levels in addition to regulating the NRF2 signaling pathway, thereby modulating the antioxidant enzymes HO‐1 and NQO‐1 [45]. Li et al. [46] discovered that the major bioactive component of *Portulaca oleracea* L., Purslane polysaccharides, has the capacity to augment the activity of N‐methyl‐N′‐nitro‐N‐nitrosoguanidine (MNNG)‐induced oxidative damage in rats with GC. This augmentation is achieved by increasing the activity of SOD, CAT, and GSH‐Px. Furthermore, it has been revealed that saffron extract Crocin has the capacity to reduce the level of oxidative stress in GC cells AGS and SGC‐7901 by activating the Nrf2 pathway. This finding suggests that Crocin has the potential to serve as an adjuvant therapy for GC [47]. A clinical randomized, double‐blind, placebo‐controlled study found that daily administration of 100 mg of saffron significantly enhanced the body's antioxidant capacity [48]. A randomized, placebo‐controlled clinical study found that daily administration of 0.5 mg resveratrol capsules significantly increased serum SOD and total antioxidant capacity (TAC), reduced serum MDA levels, enhanced antioxidant defense capabilities, and consequently improved patients' quality of life [49].

### 3.2 Anti‐Inflammatory

The present study hypothesizes that chronic inflammation is a contributing factor to the development of GC. Inflammatory factors contribute to the development and progression of GC by regulating angiogenic and adhesion molecules, tumor metastasis, and improving drug resistance [50]. It has been demonstrated that certain food and medicine homology substances possesses the capacity to elicit anti‐inflammatory effects by impeding the secretion of inflammatory mediators and modulating inflammation‐related signaling pathways, thus impeding the progression of GC [16, 30].

*Platycodon grandiflorus* polysaccharide has been shown to be effective in reducing the levels of proinflammatory cytokines, inhibiting oxidative stress, and enhancing myeloperoxidase (MPO) activity, which in turn exerts anti‐inflammatory and antioxidant effects [51]. A review of the extant literature on 42 studies suggests that honey may act as an anti‐inflammatory agent by inhibiting the action of proinflammatory factors such as TNF‐α and IL‐6 [52]. Yam polysaccharides exert antigastritic effects by reducing the expression of IL‐β, TNF‐α, and IL‐6 [53]. Vitexin, a natural flavonoid extracted from Hawthorn, has been found to possess anti‐inflammatory properties, which are attributed to its ability to attenuate 1‐methyl‐3‐nitro‐1‐nitrosoguanidine‐induced NLRP3 inflammatory vesicles. This effect contributes to the inhibition of inflammation in rats with chronic atrophic gastritis [54]. The results of the recent research indicate that cinnamon exerts an anti‐inflammatory effect by decreasing the expression levels of proinflammatory cytokines, such as TNF‐α, p‐p65, and IκBα [55]. A review reported that glycyrrhizic acid (GL) and glycyrrhizinic acid (GA) exert anti‐inflammatory effects by modulating various cytokines, including interferon‐γ, TNF‐α, interleukin‐ (IL‐) 1β, IL‐4, IL‐5, IL‐6, IL‐8, IL‐10, and IL‐12. The IL‐17 and intercellular adhesion molecules 1 and P‐selectin, in conjunction with enzymes such as inducible nitric oxide synthase (iNOS) and transcription factors such as nuclear factor‐κB (NF‐κB), signal transducer and activator of transcription (STAT)‐3 and STAT‐6, function as anti‐inflammatory agents [56]. A systematic review and meta‐analysis of randomized controlled trials found that oral curcumin supplementation exerts a marked anti‐inflammatory effect [57]. A double‐blind, randomized, placebo‐controlled clinical study found that consuming 2.5 g of turmeric juice three times weekly for 12 weeks significantly reduced levels of the tumor marker TNF‐α, thereby exerting an anti‐inflammatory effect [58].

### 3.3 Anti‐*H. pylori*

*H. pylori* infection is recognized as a significant contributing factor to the development of GC, and the range of virulence factors produced by the infection has the potential to dysregulate signaling pathways within the host, thereby lowering the threshold for carcinogenesis [59]. The infection instigates the production of key virulence factors in gastric epithelial cells, and this process is dependent on the presence of a cytotoxin‐associated gene (CagA). Besides, *H. pylori* is able to infect the human stomach and colonize the gastric mucosa. It has been demonstrated that this process elicits a gastric mucosal immune response through a variety of mechanisms, leading to inflammation [60]. *H. pylori* is the primary causative agent in 90% of GC cases, and its infection has been shown to increase the incidence of carcinogen‐induced GC. However, this increase can be significantly mitigated through the eradication of *H. pylori* vegetatively [61, 62]. Research scholars have hypothesized that some food and medicine homology substances may inhibit *H. pylori* colonization and improve *H. pylori* ‐related symptoms to a certain extent [63].

Ginger ( *Zingiber officinale* Roscoe) is a well‐known medicinal food homology substance, and the active ingredient 6‐gingerol and its derivatives have been proven to effectively inhibit the growth of *H. pylori* in vitro [64]. In addition, ginger has been demonstrated to inhibit *H. pylori* through its phenolic compounds (gingerols, shaogaol, zingerone, and phenolic acids, including gallic and cinnamic acids). The eradication rate of *H. pylori* observed in this study was 53.3% [65]. The natural compound 18β‐glycyrrhizinic acid, which is derived from licorice root, has been demonstrated to possess potent anti‐inflammatory properties. These properties were mainly reflected in the suppression of key inflammatory markers such as COX‐2, IL‐1β, iNOS, and TNF‐α. This biochemical mechanism may help protect against *H. pylori* infections by creating an unfavorable environment for the bacteria to thrive. The compound's potential to modulate inflammatory responses renders it a promising candidate for the prevention of stomach‐related infections [66]. This reduction in proinflammatory molecules can contribute to a decrease in the risk of *H. pylori* infection. Glycyrrhizin has been demonstrated to induce autophagy by inhibiting HMGB1, thereby reducing *H. pylori* infection in vitro and in vivo [67]. In addition, as demonstrated by Kim et al. [68], hesperidin effectively inhibited the expression of *H. pylori* motility‐related genes (flhA, flaA, and flgE) and adhesion‐related genes (sabA, alpA, alpB, hpaA, and hopZ). Meanwhile, downregulation of the expression of genes related to *H. pylori* replication (dnaE, dnaN, dnaQ, and holB) and transcription (rpoA, rpoB, rpoD, and rpoN) was also observed. Green fruit (olive) leaf extract is principally composed of hydrophilic compounds, including hydroxytyrosol (HT) and its glycosides. The bacteriostatic effects of these substances can significantly reduce *H. pylori* ‐induced inflammation by decreasing the secretion of AGS interleukin IL‐8 in human GC cells [69]. Kaempferia extract kaempferol inhibits the transfer of cytotoxin‐associated gene A (CagA) and vacuolar cytotoxin A (VacA), which inhibit the transfer of *H. pylori* to AGS cells, by participating in the transcription of the CagA‐injected component of the type IV secretion system (T4SS) and of the subunit protein A (SecA) of the secretion system of type V (T5SS), which is involved in VacA secretion [70]. Furthermore, Cinnamaldehyde (Cin) is one of the major active compounds isolated from *Cinnamon trees* and other cinnamon plants [71]. Li et al. [72] found that Cin exhibited excellent antibacterial activity in vitro and in vivo, and its mechanism showed that Cin could inhibit *H. pylori* by inhibiting bacterial adhesion and colonization, inhibiting biofilm formation, and regulating ATP and ROS production by targeting mainly GyrA, GyrB, AtpA, and TopA. Peng et al. [73] found that clove extract could inhibit the aberrant activation of the phosphatidylinositol‐3‐kinase‐protein kinase (PI3K‐Akt) and mitogen‐activated protein kinase (MAPK) signaling pathways, thereby treating *H. pylori* infections. Meanwhile, clove ( *Syzygium aromaticum* ) extract eugenol essential oil (EEO) has antibacterial effect against drug‐resistant *H. pylori* strains, as well as antimicrobial film and anti‐inflammatory activity [74]. A study on curcumin, the primary extract of turmeric, found that after 1 month of treatment with 500 mg of curcumin and 40 mg of famotidine daily, curcumin effectively eradicated *H. pylori* in patients [75]. Results from a clinical randomized controlled trial indicate that the addition of licorice to a clarithromycin‐based triple therapy regimen enhances the eradication rate of *H. pylori* [76].

### 3.4 Protection of Gastric Mucosa

The development of GC is frequently associated with damage to the gastric mucosa [77]. The gastric mucosa is endowed with a “gastric mucosal defence barrier” system, comprising defense factors such as the mucosal barrier, prostaglandins, and epidermal growth factor, and attack factors including oxygen free radicals and inflammatory factors. It has been demonstrated that certain food and medicine homologies substances have the capacity to form a protective film on the surface of the gastric mucosa. This film can act as a physical barrier, while stimulating the secretion of mucus by gastric mucosal cells. Furthermore, these substances can neutralize gastric acid, thereby promoting the repair of gastric mucosal epithelial cells.

Xie et al. [78] found that an aqueous extract of yams was able to inhibit the production of IL‐6, TNF‐α, and IL‐1β by reducing the expression levels of p‐NF‐κB and p‐IκB‐α, which served to suppress the inflammatory response and oxidative stress. In addition, this extract significantly upregulated Bcl‐2, downregulated Bax, and increased the secretion of growth factors, which could inhibit the proliferation of gastric mucosal cells and counteract ethanol‐induced gastric mucosal damage. *Dendrobium* polysaccharide has been shown to have the ability to impede Caspase 3 activation and PARP fragmentation. It not only regulated the expression of Bax, but also upregulated the expression of Bcl‐2 and inhibited the expression of p‐NF‐κBp65/NF‐κBp65, thus reducing the damage of ethanol to the gastric mucosa. It can also interfere with ethanol‐induced apoptosis, inhibit the loss of mucin in the gastric mucosa, and downregulate the ratio of Bax/Bcl‐2, thus exerting a protective effect on the gastric mucosa [79]. Recent research has shown that *Dendrobium officinale*'s water‐based extract can effectively alleviate acute gastric mucosal damage in rats by targeting multiple biological pathways. This protective effect works through several key actions: boosting production of protective stomach lining proteins, reducing oxidative damage, and lowering levels of inflammatory compounds like TNF‐α and IL‐6. Additionally, it suppresses critical inflammation‐related enzymes (COX‐2 and iNOS), dials down activation of p38MAPK and p65NF‐κB signaling pathways, while simultaneously increasing prostaglandin E2 (PGE2) concentrations in gastric tissues [80]. Licorice flavonoid can promote mucosal barrier repair and angiogenesis, regulate gut microbiota and SCFA metabolism, and promote epithelial cell proliferation through activation of the epidermal growth factor receptor (EGFR)/extracellular signal‐regulated kinase (ERK) pathway. This pathway plays a protective and regenerative role in the gastric mucosa [81]. Derived from cinnamon rhizomes, cinnamaldehyde can exert protective properties against gastric mucosal damage. Research has indicated that it effectively curbs autophagy, apoptosis, and ferroptosis in gastric epithelial cells by modulating key molecular players—mTOR, GSK‐3β, and NRF2—via activation of the PI3K/AKT signaling cascade. This mechanism underscores its therapeutic potential in mitigating cellular damage [82]. Furthermore, Han et al. found that cinnamon upregulated the expression of HO1 and HSP90 and also enhanced the expression of PAS and MUC, important defense molecules of the gastric mucosa, which could protect the gastric mucosa [55]. According to Wu et al., saffronin inhibited the proliferation, cycling, and metastatic ability of MNNG‐treated gastric mucosal epithelial GES‐1 cells by upregulating the Nrf2/Hippo signaling pathway [83].

Excessive secretion of gastric acid has been identified as an essential factor in the development of GC. Gastrin, a hormone that stimulates acid secretion in the stomach, has been demonstrated to serve as a potential marker for the diagnosis of this condition [84]. Zhou et al. [85] discovered that the administration of curcumin to mice carrying GC could diminish gastrin secretion, elevate gastric pH, curtail gastric acid secretion, and markedly impede the progression of GC in mice. Long et al. [86] also ascertained that curcumin was capable of regulating gastrin levels in rats with induced gastric malignancy (GML) due to rifampicin, and that it could protect against injury to the gastrointestinal tract caused by acid by targeting the IκB‐α/NF‐κB pathway.

### 3.5 Immunomodulation

The immune system plays a pivotal role in tumor surveillance and clearance [87]. At present, the prognosis of GC remains predominantly reliant on chemotherapy, even though this treatment can cause far‐reaching deleterious impacts on the quality of life of patients [88, 89]. It is noteworthy that the adverse effects of chemotherapy are not limited to cancerous cells. Indeed, normal tissues and organs are also susceptible to the toxic effects of this treatment. In severe cases, these side effects can be so severe that chemotherapy is halted, which further negatively impacts the prognosis of the patient [90]. Immunotherapy can elicit an immune response in patients with GC, resulting in the destruction of cancerous cells. In comparison with conventional therapeutic modalities, immunotherapy exhibits both robust efficacy and tolerable toxicity [91]. The combination of immunotherapy with other therapeutic modalities may emerge as a future paradigm in the management of cancer [92]. It has been demonstrated that food and medicine homology substances has the capacity to regulate the immune function of the body, enhance the activity of immune cells, and improve the body's immune response to GC cells. In addition, they are rich in nutrients, which can provide adequate nutrition and enhance the immunity of GC patients.

A number of studies have indicated that polysaccharides may impede the progression of GC by enhancing the intestinal microbiome, thereby potentiating immune system function [42]. Yam glycoproteins have been considered as potent immunomodulators [93]. They have been demonstrated to increase the production of TNF‐α, IL‐6, and NO, enhance macrophage phagocytosis and the expression of phosphorylated p38, JNK, ERK 1/2, and NF‐κB p65 proteins in peritoneal macrophages, exerting immunomodulatory activity [94]. Ganoderma lucidum polysaccharides have been demonstrated to play a significant role in immunomodulation, which can be achieved through binding to dectin‐1, TLR, or MR on monocytes, macrophages, and antigen‐presenting cells, and also through binding to CR3 on granulocytes, neutrophils, and NK cells, thus enhancing the body's immunity [95]. As reported in numerous research studies, the administration of Dendrobium officinale polysaccharides engendered a diversification of intestinal microbiota in mice. This phenomenon mainly presented an increase in the abundance of beneficial microorganisms such as rumenococci, eubacteria, and bifidobacteria, as well as a reduction in the abundance of potentially harmful bacteria. Furthermore, the polysaccharides have been observed to stimulate the aforementioned microorganisms to produce greater quantities of butyric acid. The resultant improvement in the health of the large intestine and the immune system of the mice has been well documented [96]. Research has shown that purslane polysaccharides boost immune function through multiple pathways. These compounds increase white blood cell counts, improve thymus and spleen metrics, and elevate levels of key immune markers like IL‐2, IL‐4, and TNF‐α in serum while also stimulating spleen cell growth in murine models. Similarly, amaranthus polysaccharides demonstrate immunomodulatory effects—enhancing weight gain, lymphocyte activity, white blood cell production, and cytokine secretion in rats suffering from glucocorticoid‐induced immunosuppression [46]. Recent years have seen the discovery of *Glycyrrhiza glabra* polysaccharide (GPS‐1), a licorice analogue with immunomodulatory properties. This substance can perform well in stimulating the immune system by increasing the ratio of CD3^+^CD4^+^ and CD3^+^CD8^+^ T‐lymphocyte expression, promoting dendritic cell (DC) maturation and phagocytosis, and enhancing the production of cytokines, such as IL‐4 and IFN‐γ [97]. A clinical trial involving healthy adult males demonstrated that consuming cooked coix seeds (160 g daily) for 1 consecutive week increased the abundance of *Bacteroides* species alongside CD3 + CD8+ cells, CD4+ cells, and CD4 + CD25+ cells, thereby enhancing immune function [98]. Results from a clinical randomized double‐blind controlled trial indicate that subjects demonstrated enhanced immunity following administration of *Platycodon grandiflorus* (1.5 g powder) [99].

### 3.6 Induction of Apoptosis, Inhibition of Cellular Proliferation, and Migration

#### 3.6.1 Induction of Apoptosis

Apoptosis is a classical mechanism of programmed cell death [100, 101]. Apoptosis commonly occurs in order to maintain intracellular homeostasis when cells are stimulated by factors such as inflammation and oxidative stress [102]. According to recent studies, multiple food and medicine homologies substances can activate endogenous or exogenous apoptotic pathways by regulating the expression of apoptosis‐related genes and proteins, thereby inducing apoptosis and inhibiting cell proliferation and migration in GC cells.

Astragalus is a widely used medicinal food homology substance. A soluble polysaccharide (SPS4) isolated from Astragalus can increase ROS accumulation in vivo to release cytochrome C, decrease mitochondrial membrane potential, and elevate the proapoptotic/antiapoptotic (Bax/Bcl‐2) ratio, thus exerting an obvious apoptosis‐inducing effect on the GC MGC‐803 cells [103]. A recent study has found that astragalus polysaccharide reduced the viability of SGC‐7901 or SGC‐7901/ADR cells, enhanced cell sensitivity to adriamycin, and induced cell death via the AMPK pathway [104]. Astragalus polysaccharides have further been confirmed to potentiate the antitumor efficacy of apatinib against AGS cells by inhibiting the AKR signaling pathway [105]. Furthermore, Wang et al. [106] found that 8‐paradol, a phenolic compound isolated from ginger, can induce apoptosis in human GC cells by enhancing PINK1/Parkin‐mediated mitochondrial autophagy. Curcumin, a p‐phenolic compound extracted from turmeric rhizomes, has been certified to induce apoptosis in human GC MGC‐803 cells by inhibiting the miR‐21/PTEN/Akt pathway and synergizing with PD98059 [107]. Kaempferol has been demonstrated to effectively stimulate the activation of NLRP3 inflammatory vesicles in AGS cells through the NF‐κB signaling pathway, thereby generating a signaling cascade that triggers the cleavage of caspase‐1 to ultimately release IL‐18. These processes further contributed to the apoptosis of AGS cells [108]. Kaempferol has also been shown to induce autophagy‐mediated cell death in GC cells via a pathway involving IRE 1‐JNK 1‐mediated Bcl‐2–beclin‐1 and HDAC/G9 [109]. The majority of the active components in licorice have been identified to promote apoptosis in GC cells, with glycyrrhizic acid being particularly noteworthy. Indeed, recent research has demonstrated that glycyrrhizic acid induces apoptosis in GC cells by upregulating the expression levels of Bax, cleaved PARP, and cysteinyl asparaginogen‐3, 8, and 9 [110]. Ginkgo lactone is a sesquiterpenoid from *Ginkgo biloba* , family Ginkgoaceae. The results of in vitro experiments have demonstrated that ginkgo (bilberry) can effectively inhibit the growth of AGS and induce AGS cell death through nuclear damage and apoptosis [111]. Ginkgolic acid has been likewise evidenced to induce apoptosis in GC cells in a dose‐dependent manner. This process involves the activation of caspases 9 and 3, as well as poly (ADP‐ribose) polymerase (PARP). Concurrently, the expression levels of Bcl‐2 and Bcl‐xl have been observed to decrease. The elevated levels of Bax and Bad can also inhibit the signaling and transcriptional activator of transcription 3/Janus kinase 2 (Stat 3/JAK 2) signaling pathway activation [112]. Cinnamaldehyde, the key bioactive compound found in cinnamon, has demonstrated a dose‐dependent suppression of the Jak 2/Stat 3 signaling cascade. This natural compound effectively lowers the Bcl‐2/Bax protein ratio, triggering programmed cell death in gastric carcinoma cells. Research confirms cinnamon's medicinal properties operate through these precise molecular mechanisms [113]. In vivo and in vitro studies have indicated that citrus‐derived polymethoxyflavone can induce apoptosis through the upregulation of RARβ, which is the value added of GC cells [114]. A sesquiterpene lactone compound extracted from chicory, costunolide (Cos), has been reported to significantly inhibit the growth of HGC‐27 and SNU‐1 cells and to block the cell cycle in the G2/M phase. In addition, this compound can induce apoptosis through the promotion of cytosolic ROS, the inhibition of the ROS‐AKT/GSK3β signaling pathway, and the activation of autophagy in prodeath cells. The process of apoptosis is induced by the inhibition of the ROS‐AKT/GSK3β signaling pathway and the activation of prodeath cell autophagy [115]. The *lilium lancifolium* saponins of curdlan is a saponin compound extracted from *lilium lancifolium* saponins. It has been demonstrated that this compound induces apoptosis by upregulating the expression of the proapoptotic protein Bax and downregulating the expression of the antiapoptotic protein Bcl‐2 [29]. Quercetin, present in numerous substances that serve both medicinal and dietary purposes, induces apoptosis in GC cells by increasing ROS and enhancing the expression of proapoptotic proteins Bad, Bax, and Bid [116].

#### 3.6.2 Inhibition of Cell Proliferation and Migration

A significant challenge in the realm of tumor suppression and cancer treatment is the capacity of tumors to invade and metastasize. Tumor invasion and metastasis have been identified as significant contributors to the decline in cancer patients' quality of life and are even the primary causes of mortality in this demographic [117]. Fortunately, multiple food and medicine homology substances possess the capacity to impede the proliferation of GC cells by modulating cycle‐related proteins and pathways associated with cell proliferation.

Saffronin, a key bioactive compound found in saffron, has been shown to suppress epithelial–mesenchymal transition while curbing the invasive and migratory behavior of GC cells. This effect occurs through the upregulation of KLF5, HIF‐1α, and miR‐320 signaling pathways in both GC tissues and cellular environments [118]. In addition, saffronin can inhibit the growth of GC cells by increasing the expression of AKR1Cs and decreasing the expression of CHI3L1 or TMP4 [43, 119]. As well, saffronic acid may impede the processes of angiogenesis and metastasis in GC cells by decreasing the expression of the Sonic hedgehog gene [120]. Therefore, saffron could hold promise as a potential anticancer agent. The flavonoid isoglycyrrhizin (ISL), a key bioactive compound in licorice, has demonstrated the ability to suppress glucose‐regulated protein 78 (GRP78) activity—effectively curbing cancer stem cell traits, the expression of stem cell‐related proteins, and the activation of cancer‐associated fibroblasts. These findings highlight its potential therapeutic value in oncology [121]. In addition, ISL has been demonstrated to inhibit gastric tumor growth in a xenograft animal study. 18 β‐Glycyrrrhetinic acid, a licorice compound, has been proven to inhibit the proliferation of GC cells by targeting the MAPK signaling pathway and regulating cytokines such as MRPL35, COPS5, TP53, thereby playing an anti‐GC role [122, 123]. Gong et al. [124] found that glycyrrhizinone exerts anti‐GC effects in both in vivo and in vitro models. The study has revealed that the compound could inhibit gastric tumor growth by targeting the vascular endothelial growth factor receptor‐2 (VEGFR‐2) and blocking the PI3K/AKT and MEK/ERK signaling pathways in VEGF‐stimulated MKN‐45 cells. Subsequently, the proliferation, migration, and invasion of GC cells MKN‐45 were further inhibited. Platycodonin D, a triterpenoid saponin compound isolated from *Platycodon grandiflorus* , has been shown to regulate survivin protein expression in both in vivo and in vitro models through the action of microRNA‐34a [125]. Furthermore, *Platycodon grandiflorus* polysaccharide has been shown to inhibit the growth and proliferation of GC cells by promoting the degradation of proto‐oncogene protein (c‐Myc), leading to the inactivation of the p21/CDK2‐Cyclin E pathway, and significantly decreasing the activity of NUGC3 and NZ521 GC cells, which can in turn inhibit the growth and proliferation of GC cells [126]. Curcumin, a polyphenolic compound found in turmeric, has been shown to downregulate key genes and proteins in the PI3K signaling pathway, such as PI3K, Akt, and mTOR, thereby inhibiting the proliferation of human GC cells (AGS) [127]. In addition, curcumin has been reported to impede the invasion and proliferation of GC cells by targeting oncogenic factors, such as p23 and human epidermal receptor 2 (including *H. pylori*) [128]. In the study conducted by Wang et al. [129], it was discovered that the isopentenylated flavonoid sanguinarone, isolated from the root bark of mulberry, can inhibit the proliferation and growth of human GC cells by downregulating c‐Myc in the cells. The total saponins present in curdlan have been identified to inhibit the proliferation of GC cells by suppressing the level of proliferating cell nuclear progenitor (PCNA) and increasing the level of p21. Isorhamnetin (ISO) extracted from sea buckthorn has displayed potent antiautophagic and antiproliferative function against GC cells under hypoxic conditions. It exerts these actions by specifically inhibiting PI3K, thereby disrupting the PI3K‐AKT–mTOR signaling cascade [130]. Porinic acid, a triterpenoid extracted from Poria cocos, is prominent in its capacity to inhibit the invasion and migration of human GC cells AGS and MKN‐28. This effect is attributed to the ability of porinic acid to inhibit the expression of EMT‐related proteins, such as E‐cadherin, N‐cadherin, and Vimentin, and to decrease the expression of metastasis‐related proteins, including MMP‐2, MMP‐9, and TIMP‐1 [131]. Xie et al. [132] also found that the alcoholic extract of Poria cocos could inhibit the proliferation of human GC cells MKN45 by suppressing the signaling pathways that can be downregulated by MAPK and PI3K‐Akt. Lobetyolin, a principal active ingredient of *Codonopsis pilosula* , has been shown to possess antineoplastic properties, including the capacity to inhibit GC. Mechanistically, lobetyolin exerts its antineoplastic effects by enhancing the protein expression of p‐c‐Myc at Thr 58, decreasing the protein expression of p‐AKT at Ser 473 and p‐GSK 3 β at Ser 9, and modulating the AKT/GSK 3 β/c‐Myc signaling pathway to reduce the expression of ASCT 2 in GC cells [133]. Hesperidin, a citrus flavonoid, is present in significant quantities in the medicinal substance orange peel. It has been demonstrated that this substance can reverse N‐methyl‐N‐nitro‐N‐nitroguanidine‐induced GC by promoting autophagy and PI3K/AKT pathway activity [134]. Resveratrol, the primary extract from mulberries, serves as a representative chemopreventive anticancer compound. It directly interacts with PIM‐1 to inhibit the proliferation and migration of SNU‐601 GC cells [135].

## 4 Summary and Outlook

Through an extensive review of the literature, this study identifies licorice as a remarkable food and medicine homology substances, demonstrating significant anti‐*H. pylori*, protection of gastric mucosa, immunomodulation, potential resistance modulation, as well as the ability for induction of apoptosis, inhibition of cellular proliferation, and migration. In terms of pharmacological mechanisms, food and medicine homology substances mainly regulate cell signaling pathways and influence the proliferation, apoptosis, and migration of GC cells. Their modulation of the immune system can enhance the recognition and elimination of cancer cells by immune cells. In addition, they can inhibit the progression of GC by improving the tumor microenvironment. These components play a crucial role in the preventive stage and serve as adjunctive therapies in clinical treatment, helping to mitigate the toxic side effects of radiotherapy and chemotherapy, improve patients' nutritional status, and enhance their quality of life. Thus, it provides a promising integrated dietary intervention strategy for the prevention and treatment of GC. Concurrently, these substances also possess antioxidant capacity, thereby diminishing the cellular damage caused by free radicals to the gastric mucosa. Consequently, these mechanisms synergistically contribute to reducing the risk of GC. In experimental studies, both cellular and animal experiments have provided strong evidence for the effects of food and medicine homology substances on GC. The investigation revealed that a range of food and medicine homology substances extracts were capable of significantly inhibiting the proliferation of GC cells and inducing their apoptosis in vitro. Furthermore, evidence has been provided to demonstrate the capacity of these substances to inhibit tumor growth in animal models. Several small‐scale clinical trials have indicated that the reasonable intake of specific food and medicine homology substances can improve the symptoms and quality of life in GC patients. Furthermore, these substances can play an adjunctive role to traditional treatments, such as chemotherapy, and alleviate the adverse reactions in patients. The prolonged use of food and medicine homology substances may potentially entail certain risks. Excessive long‐term intake of specific components has the potential to trigger toxic reactions; for instance, prolonged consumption of ginger may cause gastrointestinal discomfort or interact with anticoagulant medications, increasing the risk of bleeding. Furthermore, the long‐term use of such substances may lead to interactions between their active constituents and conventional pharmaceuticals, thereby affecting therapeutic efficacy or heightening the risk of adverse reactions. Consequently, when employing these substances, it is imperative to strictly adhere to traditional dosage guidelines and prescribed treatment durations. Food and medicine homology substances may exhibit issues such as low water solubility and poor oral bioavailability. Nanomedicine delivery systems can be employed, wherein nanocarriers shield the encapsulated molecule from lysis and pH alterations, enhance its solubility, and facilitate sustained drug release to target cells. Gelatin‐based nanocarriers effectively enhance drug absorption in the gastrointestinal tract and improve its in vivo distribution through their biocompatibility, mucosal adhesion, and controllable release properties, thereby achieving targeted delivery.

The review investigates the potential of food and medicine homology substances to prevent and treat GC through multitargeted, holistic regulation of the organism. The properties of these substances include antioxidant, anti‐inflammatory, and anti‐ *H. pylori* effects, with the ability to protect the gastric mucosa, modulate immunity, induce cancer cell apoptosis, and inhibit proliferation and metastasis (Table 1). These ingredients have been demonstrated to inhibit carcinogen formation, enhance immunity, and reduce cancer risk. As adjunctive therapeutic components, they have been demonstrated to have significant benefits. For instance, hawthorn and dried tangerine peel have been shown to improve appetite in chemotherapy patients and alleviate indigestion, while astragalus and goji berries have been demonstrated to regulate immunity and enhance quality of life. The food and medicine homology substances is naturally safe, with minimal side effects, and they synergize with conventional treatments to mitigate toxic side effects. In essence, the bioactive compounds present in medicinal foods provide both scientific rationale and practical pathways for the prevention and treatment of GC. Network pharmacology techniques may be employed to screen for active components and targets in substances with dual medicinal and food properties and in diseases. The integration of traditional wisdom with modern nutrition and pharmacology not only enables daily cancer prevention but also serves as an effective adjunct to integrated Chinese and Western medical treatments. This approach is conducive to the achievement of safer, personalized health management objectives. Thus, there is an urgent need to break through the bottleneck of existing research by developing functional foods, health products, or auxiliary therapeutic agents based on medicinal and food ingredients to enhance their bioavailability and clinical efficacy. The main objective is twofold: firstly, to overcome the limitations of extant research in this field; and secondly, to promote the wide application of medicinal and food ingredients in clinical practice.

**TABLE 1:** Food and medicine homology substances and their bioactive components with prevention and treatment of GC.

Pharmacological action	Food and medicine homology	Active ingredient	References
Antioxidant	Mulberry, Coix seed, Polygonatum, Chinese bellflower, Ganoderma, *Dendrobium officinale*, Purslane	Oligofructose, Resveratrol, *Polygonatum sibiricum* polysaccharides, *Ganoderma lucidum* polysaccharide, *Dendrobium officinale* polysaccharides, Purslane polysaccharides, Crocin	[37, 38, 39, 40, 41, 42, 43, 44, 45, 46, 47, 48, 49]
Anti‐inflammatory	Chinese bellflower, Honey, Yam, *Crataegus Pinnatifida* , Cassia	*Platycodon grandiflorus* polysaccharide, Yam polysaccharides, Vitexin	[51, 52, 53, 54, 55, 56, 57, 58]
Anti‐*H. pylori*.	Ginger, Cassia, Licorice, Orange peel, Olive leaf, Kaempferia, Clove	6‐gingerol and its derivatives, 18β‐glycyrrhizinic acid, Glycyrrhizin, Hesperidin, Green fruit (olive) leaf extract, Kaempferol, Cinnamaldehyde, Eugenol essential oil	[64, 65, 66, 67, 68, 69, 70, 71, 72, 73, 74, 75, 76]
Protection of gastric mucosa	*Dendrobium officinale*, Yam, Cassia, Licorice, Astragalus, Turmeric, Saffron	Yam water extract, *Dendrobium* polysaccharide, *Dendrobium officinale* water extract, Licorice flavonoid, Cinnamaldehyde, Saffronin, Ginger aqueous extract, Curcumin	[78, 79, 80, 81, 82, 83], [85, 86]
Immunomodulation	Yam, Ganoderma, *Dendrobium officinale*, Purslane, Licorice	Yam glycoproteins, Ganoderma lucidum, *Dendrobium officinale* polysaccharides, Purslane polysaccharides, *Glycyrrhiza glabra* polysaccharide	[42], [93, 94, 95, 96, 97, 98, 99]
Induction of apoptosis, inhibition of cellular proliferation, and migration	Mulberry, Chinese bellflower, Ganoderma, *Crataegus pinnatifida* , Ginger, Licorice, Orange peel, Astragalus, Turmeric, Ginkgo, Chicory, Saffron, *Lilium lancifolium* , Sea buckthorn, Poria, *Codonopsis pilosula*	*Astragalus polysaccharide*, 8‐paradol, Urcumin, Kaempferol, Glycyrrhizic acid, Ginkgo lactone, Ginkgolic acid, Cinnamaldehyde, Polymethoxyflavone, Costunolide, *Lilium lancifolium* saponins, Saffronin, Flavonoid isoglycyrrhizin, 8 β‐Glycyrrrhetinic acid, Glycyrrhizinone, Platycodonin D, *Platycodon grandiflorus* , Curcumin, Sorhamnetin, Porinic acid, Lobetyolin, Hesperidin	[43], [103, 104, 105, 106, 107, 108, 109, 110, 111, 112, 113, 114, 115, 116], [118, 119, 120, 121, 122, 123, 124, 125, 126, 127, 128, 129, 130, 131, 132, 133, 134, 135]

## Author Contributions

**Zhengyang Hao**, and **Xiangjun Chen:** writing – original draft preparation; **Zhengyang Hao, Wenqian Tang**, and **Ruipeng Wu:** writing – review and editing; **Bei Xue:** formal analysis; **Jingzhe Chen**, and **Yidan Zhang:** software; **Shaokang Wang:** supervision, project administration, and funding acquisition. All authors have read and agreed to the published version of the manuscript.

## Conflicts of Interest

The authors declare no conflicts of interest.

## Data Availability

The data that supports the findings of this study are available in the Supporting Information of this article.
